# Long-term immunogenicity of a single-dose live recombinant chimeric Japanese encephalitis vaccine in adults

**DOI:** 10.1093/jtm/taaf006

**Published:** 2025-01-21

**Authors:** Deborah J Mills, Narayan Gyawali, Nirupama A Nammunige, Christine Mills, Gregor J Devine, Colleen L Lau, Luis Furuya-Kanamori

**Affiliations:** Dr Deb The Travel Doctor, Travel Medicine Alliance, Brisbane, Australia; UQ Centre for Clinical Research, Faculty of Health, Medicine, and Behavioural Sciences, The University of Queensland, Herston, Australia; Mosquito Control Laboratory, QIMR Berghofer Medical Research Institute, Brisbane, Australia; Mosquito Control Laboratory, QIMR Berghofer Medical Research Institute, Brisbane, Australia; Dr Deb The Travel Doctor, Travel Medicine Alliance, Brisbane, Australia; Mosquito Control Laboratory, QIMR Berghofer Medical Research Institute, Brisbane, Australia; UQ Centre for Clinical Research, Faculty of Health, Medicine, and Behavioural Sciences, The University of Queensland, Herston, Australia; UQ Centre for Clinical Research, Faculty of Health, Medicine, and Behavioural Sciences, The University of Queensland, Herston, Australia

**Keywords:** Arbovirus, flavivirus, immunity, non-endemic, persistence

## Abstract

**Background:**

Japanese encephalitis virus is a leading cause of viral encephalitis in Asia, with high case-fatality rate and morbidity. Although the live recombinant Japanese encephalitis chimeric vaccine (Imojev®) offers strong initial immunity, data on long-term efficacy beyond 5 years remain limited.

**Methods:**

We conducted a cross-sectional study on adults vaccinated with Imojev® at a specialist travel clinic in Brisbane, Australia. Participants were stratified based on the time since vaccination: 2–5 years and >5 years. Neutralizing antibody titres were measured using the plaque reduction neutralization test (PRNT_50_), with titres ≥10 indicating seropositivity.

**Results:**

Of the 103 participants, 47 were vaccinated 2–5 years prior and 56 were vaccinated ≥5 years prior to enrolment. All participants vaccinated within 5 years remain seropositive, whilst 52 of 56 (92.9%) vaccinated ≥5 years ago were seropositive. Four participants (7.1%) were seronegative post-vaccination, with time since vaccination ranging from 5 to 9 years. These seronegative individuals were vaccinated a median of 9.2 years ago, compared to 5.1 years for seropositive participants (*P*-value = 0.037). Aside from time since vaccination, no other factors (e.g. age, sex) were associated with seronegativity.

**Conclusions:**

Imojev® provides durable immunity, with seropositivity exceeding 90% up to 10 years post-vaccination. However, waning immunity in a small proportion of individuals suggests that booster doses may be beneficial for high-risk travellers vaccinated over 5 years ago.

## Introduction

Japanese encephalitis virus (JEV) is endemic to parts of Asia and the Torres Strait region of Australia, where it remains the leading cause of viral encephalitis, responsible for approximately 100 000 clinical cases and 25 000 deaths annually.^1^ Japanese encephalitis (JE) has a high case-fatality rate of around 30%,^2^ and survivors often suffer from long-term neurological sequelae, affecting 30–50% of symptomatic patients.^3^ Whilst no specific antiviral treatment exists for JE, vaccination is a critical measure for disease prevention and outbreak control.^4^

Currently, there are three primary vaccines available to protect against JE: (i) the live attenuated SA14–14-2 strain, primarily used in China (marketed as CD.JEVAX®); (ii) inactivated Vero cell-derived vaccines (marketed as Ixiaro® or JEspect®) and (iii) live recombinant chimeric vaccines (marketed as ChimeriVax™ or Imojev®).^5^^,^^6^ In non-endemic countries, JE vaccines are recommended for travellers spending 1 month or more in JE-endemic areas.^6–9^ Vaccination is also advised for shorter-term travellers (<1 month) who may be at increased risk due to factors such as travel to endemic areas during the wet season, ongoing travel to high-risk areas, extensive outdoor activities, or staying in accommodation lacking air-conditioning, screens, or bed nets.^7^^,^^9^^,^^10^

For immunocompetent adults (aged ≥18 years) in non-endemic regions, two vaccine options and schedules are recommended (depending on the availability of the vaccines):

- Two intramuscular (IM) doses of inactivated Vero cell-derived vaccine, administered 28 days apart, with a booster dose recommended after 1–2 years for individuals with ongoing exposure risk^7^^,^^9^;
- A single subcutaneous (SC) dose of the live recombinant chimeric vaccine, with booster doses currently not recommended.^7^

Both vaccination schedules are safe^11^ and provide adequate short-term protection,^12^ but evidence on their long-term efficacy, particularly beyond 5 years, remains limited. To date, only one study in adults has assessed long-term immunity for the live recombinant chimeric vaccine. Nasveld *et al.* followed up 93 Australian Defence Force personnel who received the live recombinant chimeric vaccine and found that seropositivity rates declined over time—from 100% at 6 months, to 92% at 3 years, and 87% at 5 years post-vaccination.^13^

The lack of robust data on the longevity of protection from the live recombinant chimeric vaccine is particularly notable, as booster doses are currently not recommended for adults. In endemic regions, individuals may experience natural boosting of immunity through re-exposure to JEV, which can prolong the duration of protective antibody levels. A study on long-term expatriates residing in Thailand demonstrated seropositivity to multiple arboviruses, including JE, likely reflecting ongoing exposure and boosting in an endemic environment.^14^ In contrast, in non-endemic settings, vaccine-induced immunity relies solely on the longevity of the immune response elicited by vaccination. Given the potential risk of JE for travellers from non-endemic regions who were vaccinated ˃5 years prior, this study seeks to address the current gap in evidence. We aimed to assess the long-term protection provided by the JE live recombinant chimeric vaccine in adults from non-endemic regions.

## Methods

### Study setting and study population

Participants were enrolled from a specialist travel medicine clinic, Dr Deb The Travel Doctor in Brisbane, Australia between 29 November 2022 and 1 August 2024. The clinic has administered over 2600 doses of JE live recombinant chimeric vaccine from 2013 to 2020. Eligible participants were identified through electronic medical records, and those who met the inclusion criteria were contacted and invited to participate.

Eligibility criteria included: adults (aged ≥18 years); received live recombinant chimeric vaccines >2 years ago; and not lived in JEV-endemic areas for ˃12 months. Participants were excluded if they had a history of JEV infection or had received a JE booster dose after the initial vaccination.

Written informed consent was obtained from all participants before enrolment. The study was approved by the Australian National University Human Research Ethics Committee (2022/433), ratified by the University of Queensland Human Research Ethics Committee (2022/HE002356) and the QIMR Berghofer Human Research Ethics Committee (P3852).

### Sample size

Our target enrolment was 100 participants, evenly divided between those who received the JE live recombinant chimeric vaccine 2–5 years prior and those vaccinated ˃5 years ago. The sample size calculation was based on the precision of estimating a single proportion—specifically, the seropositivity rate in participants vaccinated ˃5 years ago. In the study by Nasveld *et al*., seropositivity rates ranged from 87% to 93% amongst individuals vaccinated 2–5 years prior.^13^ Based on these findings, we conservatively estimated an 80% seropositivity rate for those vaccinated ˃5 years ago. This sample size was projected to yield a 95% confidence interval (CI) ranging from 66.3% to 90.0%.

### Data collection

At enrolment, participants completed a questionnaire to collect demographic data (age, sex), vaccination history (date of JEV vaccination), and medical history (including comorbidities, history of travel and other flavivirus infections). Information on comorbidities and vaccine history was collected through self-reporting and confirmed by medical records.

Blood samples (10 mL) were collected in BD vacutainer serum tubes for serological testing. Samples were centrifugated at 2500 × *g* for 10 min at room temperature and the separated serum was stored at −20°C until transported to the QIMR Berghofer for laboratory analysis. Participants were informed of their individual results.

### Laboratory analysis

JEV neutralizing antibodies were measured by the plaque reduction neutralization 50% (PRNT_50_) method, as described in our previous study.^15^ In summary, BHK-21 cells (1.5 × 10^5^ cells per well) were seeded in 24-well plates and cultured for 24 h. Serum samples were inactivated at 56°C for 30 min and serially diluted four-fold from 1:10 to 1:640. These were mixed with JEV (Nakayama strain, kindly provided by Dr Wenjun Liu, Australian Defence Force Malaria and Infectious Disease Institute), and incubated for 60 min before being applied to the BHK-21 monolayer cells. After 2 h incubation of serum plus virus mixture on BHK-21 cell monolayer, an overlay of equal proportion of carboxymethyl cellulose medium and ds-RPMI (pH 7.6) was added. Following an incubation for 3 days at 37°C, cells were stained using 0.2% crystal violet-formaldehyde-methanol solution, and resulting virus plaques forming units (pfu) were counted. The PRNT_50_ titre was defined as the reciprocal of the highest serum dilution that resulted in a 50% or greater reduction in pfu of virus control.

### Statistical analysis

Descriptive statistics were used to summarize participants' characteristics, including age, sex, time since vaccination and comorbidities. The primary outcome, JEV seropositivity defined as PRNT_50_ titres ≥10, was reported as the proportion of participants who were seropositive at the time of serum collection. Participants were grouped them based on the time since their vaccination, and geometric mean titres (GMTs) were calculated for each year post-vaccination. Comparisons between seropositive and seronegative participants were made using the Mann–Whitney U test for continuous variables (e.g. time since vaccination) and the chi-square test for categorical variables (e.g. age, sex, comorbidities).

All tests were 2-tailed, and a *P*-value < 0.05 was deemed statistically significant. Statistical analyses were conducted using Stata MP version 14 (StataCorp, College Station, Texas).

## Results

### Participants’ characteristics

Around 800 eligible travellers were contacted, of which 103 agreed to participate and were enrolled in the study. Of the 103 participants, 47 (45.6%) and 56 (54.4%) received JE live recombinant chimeric vaccination 2–5 years and > 5 years ago, respectively. Overall, participants received JE vaccine between 2.3 and 10.8 years prior to enrolment [median: 5.3 years, interquartile range (IQR): 4.4–8.6 years] (Supplementary Material S1).

Half (50.5%) of the participants were females, the median age at vaccination was 47.5 years (IQR 37.4–56.8 years, range 12.6–79.4 years), and the median age at enrolment was 54.4 years (IQR 44.6–62.6 years, range 21.2–82.8 years). Over half of the participants (53.4%) reported at least one comorbidity, with the most common being dyslipidaemia (17.5%), hypertension (13.6%), asthma or chronic obstructive pulmonary disease (9.7%), and anxiety or depression (8.7%) (Table 1).

**Table 1:** Demographic characteristics and medical history of the included participants

	Number (%) or [IQR]
Demographics	
Sex	
Female	52 (50.5)
Male	51 (49.5)
Median age (in years) at vaccination [IQR]	47.5 [37.4–56.8]
Age groups at vaccination (in years)	
<20	4 (3.9)
20–39	26 (25.2)
40–59	56 (54.4)
>60	17 (16.5)
Median age (in years) at enrolment [IQR]	54.4 [44.6–62.6]
Age groups at enrolment (in years)	
<40	20 (19.4)
40–59	45 (43.7)
60–79	37 (35.9)
>80	1 (1.0)
Comorbidities^a^	55 (53.4)
Dyslipidaemia	18 (17.5)
Hypertension	14 (13.6)
Asthma/COPD	10 (9.7)
Anxiety/depression	9 (8.7)
Musculoskeletal condition	8 (7.8)
Gastroesophageal reflux disease	7 (6.8)
Thyroid diseases	6 (5.8)
History of cancer	3 (2.9)
Diabetes mellitus	3 (2.9)
Coronary artery disease	2 (1.9)
Obesity	2 (1.9)
Neurological disease	2 (1.9)
Renal disease	1 (1.0)
Other comorbidities	7 (6.8)

^a^26 participant reported more than one comorbidity.

COPD, chronic obstructive pulmonary disease; IQR, interquartile range.

### Long-term immunogenicity

**Figure 1:**
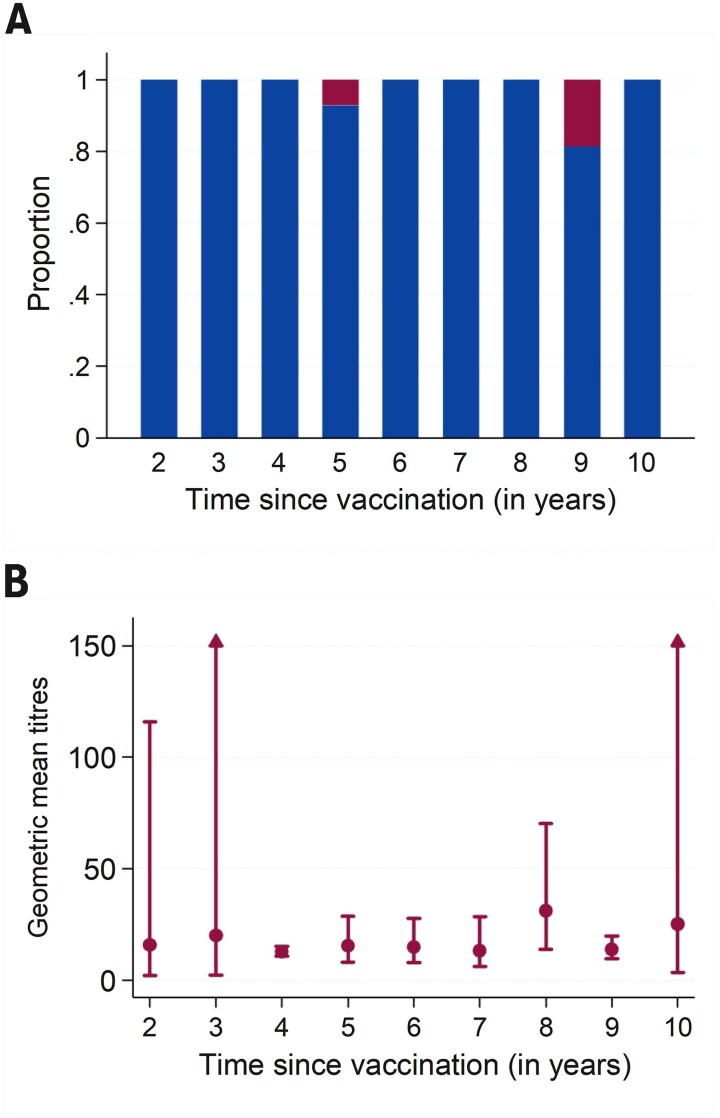
(A) Proportion of participants who were seropositive (blue) and seronegative (red); and (B) geometric mean titres and 95% confidence intervals by time since primary vaccination with live chimeric Japanese encephalitis vaccine

Of the 103 participants, 99 (96.1%) were JEV seropositive (neutralizing antibody titres >10). All 47 participants vaccinated 2–5 years ago were seropositive, whilst 92.9% (52 out of 56) of those vaccinated >5 years ago remained seropositive. The four seronegative participants had received the vaccine 5 years (n = 1) or 9 years prior (n = 3) (Figure 1A). The GMTs remained stable over time, fluctuating between 12.7 at 4 years and 31.1 at 8 years post-vaccination (Figure 1B; Supplementary Material S2).

Amongst the four seronegative participants, none had uncontrolled or severe comorbidities at the time of serum collection. However, one participant was subsequently diagnosed with non-Hodgkin lymphoma.

 

– Participant 1: A 30-year-old female with no comorbidities at the time of vaccination, and was seronegative 5 years post-vaccination. The participant was diagnosed with a Stage 1A non-Hodgkin lymphoma 9 months after her negative serology test.
– Participant 2: A 28-year-old male with no comorbidities was seronegative 9 years post-vaccination.
– Participant 3: A 35-year-old female on selective serotonin reuptake inhibitors for mood disorder, tested negative 9 years post-vaccination.
– Participant 4: A 46-year-old male with no comorbidities was seronegative 9 years post-vaccination.

No significant demographic characteristics (e.g. age, sex) or medical history (e.g. comorbidities) were identified amongst JEV seronegative participants. However, the median time since vaccination was significantly longer in seronegative participants (9.2 years; IQR: 7.5–9.4 years) compared to seropositive participants (5.1 years; IQR: 4.4–8.3 years) (*P-value* = 0.037).

## Discussion

Our study provides valuable insights into the long-term immunogenicity of the JE live recombinant chimeric vaccine. Although its use is currently limited to a few countries (i.e. Australia, South Korea, Thailand), its adoption is expected to expand in the near future.^5^ With an overall seropositivity rate exceeding 95%, our findings align closely with previously reported seropositivity rates at shorter intervals,^16^ demonstrating that the great majority of individuals had sustained immunity up to 10 years post-vaccination. In participants vaccinated within the last 2–5 years, the seropositivity rate was 100%, whereas amongst those vaccinated over 5 years ago, 92.8% of individuals remained seropositive.

The median time since vaccination was significantly longer in seronegative participants (9.2 years) than in seropositive ones (5.1 years), suggesting a possible waning of immunity. This decline, though affecting only a minority, raises the question of whether booster doses should be considered for travellers at high risk of JE exposure if their initial vaccination was over 5 years ago. JE live recombinant chimeric vaccine has been shown to provide robust initial immunogenicity, with studies indicating seroconversion rates of >97% within 1- and 2-months post-vaccination.^12^ Despite the initial strong response, evidence from Nasveld *et al.,*^13^ a modelling study by Desai *et al*.,^17^ and the current study, indicate that immunity wanes in some individuals after 5 years. Given the relatively low incidence of symptomatic JE in exposed populations (⁓1 in 200),^18^^,^^19^ it is challenging to gauge the clinical implications of PRNT_50_ titres falling below the nominal protective thresholds in asymptomatic individuals. Although boosters are not currently recommended for adults, children who receive JE live recombinant chimeric vaccine at between 9 months and 18 years of age are advised to receive a booster 1–2 years after the initial dose.^7^ Booster doses may also benefit adults vaccinated over 5 years ago, particularly those with weakened immunity or who plan to travel to high-risk areas. Few studies have demonstrated the efficacy and safety of JE booster doses in adults,^16^^,^^20^^,^^21^ and further research is needed to determine the optimal schedule (time between primary vaccination and booster dose).

The protective threshold of PRNT_50_ titres ≥10 is widely used in clinical trials to indicate JE immunity, but it remains unclear whether individuals with lower titres are entirely unprotected.^22^ Given the variable severity of JE^23^—ranging from asymptomatic to fatal encephalitis—it is possible that those with low or undetectable antibodies may still mount an immune response if exposed to JEV, possibly reducing their likelihood of symptomatic infection. However, further research on potential vaccine failure rates and the protective value of PRNT_50_ thresholds are needed, especially considering that a single documented case of JE in a vaccinated (JE type vaccine, route and dosage not reported) child (sex and age not reported) suggests that failures, though rare, can occur.^24^

The emergence of some JEV genotypes, such as genotype IV (G-IV), in the recent outbreak in Australia, poses another consideration for JE vaccination strategies.^25^ Current vaccines, developed against genotype III (G-III), have shown varying efficacy against newer strains,^26^ with studies suggesting lower neutralizing antibody levels against G-IV.^27^ Although G-IV remains rare, continued surveillance and potential vaccine adjustments may be necessary to ensure comprehensive protection as JEV evolves.

This study offers valuable data with extended follow-up of up to 10 years, a perspective that is less frequently documented in JE vaccine studies. However, certain limitations should be noted. Our study population was recruited from a specialist travel medicine clinic, which may not fully represent the broader population of Australian travellers. The clinic is likely to attract individuals with more complex travel itineraries or specific occupational health requirements. Furthermore, a convenience sampling method was utilized which may limit the generalisability of our findings, particularly those with lower-risk travel profiles. However, it aligns with the target population for JE vaccination recommendations—travellers visiting high-risk or endemic areas. The COVID-19 pandemic disrupted travel, so there were fewer travellers available to recruit for the 2–5-year post-vaccination group.

In conclusion, our study indicates that whilst JE vaccination confers long-term immunity, a small proportion of individuals vaccinated over 5 years ago may experience waning protection. Given that 7.3% of JE live recombinant chimeric vaccine recipients were seronegative beyond 5 years, healthcare providers of travellers with potential immune deficiencies or those at high risk of JEV exposure should discuss the risk and benefits of a booster.^28^ Further studies are needed to establish definitive booster guidelines and assess immunity against emerging JEV genotypes.

## Supplementary Material

jelli_sup_material_taaf006
