# Supplementary material for: Long-term immunogenicity of a single-dose live recombinant chimeric Japanese encephalitis vaccine in adults
Source: J Travel Med. 2025 Jan 21;32(2):taaf006. doi: 10.1093/jtm/taaf006 (PMC11896838; doi:10.1093/jtm/taaf006)
Supplement: jelli_sup_material_taaf006 [file jelli_sup_material_taaf006.docx]

**Supplementary material**

**S1.** Number of seropositive (blue) and seronegative (red) participants by time since primary vaccination with live chimeric Japanese encephalitis vaccine

|  |
| --- |

**S2.** Neutralising antibody titre levels by time since primary vaccination with live chimeric Japanese encephalitis vaccine

| **** |
| --- |
